# Brain radiotherapy, tremelimumab-mediated CTLA-4-directed blockade +/− trastuzumab in patients with breast cancer brain metastases

**DOI:** 10.1038/s41523-022-00404-2

**Published:** 2022-04-19

**Authors:** David B. Page, Kathryn Beal, Stefanie N. Linch, Kateri J. Spinelli, Micaela Rodine, Darragh Halpenny, Shanu Modi, Sujata Patil, Robert J. Young, Thomas Kaley, Taha Merghoub, David Redmond, Phillip Wong, Christopher A. Barker, Adi Diab, Larry Norton, Heather L. McArthur

**Affiliations:** 1 Providence Cancer Institute, Earle A. Chiles Research Institute, 4805 NE Glisan St., Portland, OR 97213 USA; 2 Memorial Sloan Kettering Cancer Center, 1275 York Avenue, New York, NY 10065 USA; 3 Weill Cornell Medicine, 1300 York Avenue, New York, NY 10065 USA; 4 The University of Texas MD Anderson Cancer Center, 1515 Holcombe Blvd, Houston, TX 77030 USA; 5 University of Texas Southwestern, 5323 Harry Hines Blvd, Dallas, TX 75235 USA

**Keywords:** Cancer, Cancer immunotherapy

## Abstract

Breast cancer brain metastases (BCBM) are a common and devastating complication of metastatic breast cancer with conventional systemic therapies demonstrating limited effectiveness. Consequently, radiotherapy (RT) ± surgery remains the cornerstone of BCBM management. Because preclinical and clinical evidence indicate that immune checkpoint blockade (ICB) may synergize with RT to promote systemic tumor regression, we explored the safety and efficacy of RT and concurrent tremelimumab-mediated cytotoxic T-lymphocyte associated protein 4 (CTLA-4) ICB with tremelimumab ± HER2-directed therapy with trastuzumab for BCBM. Eligible patients had BCBM indicated for brain RT. A Simon two-stage design was adopted to evaluate the efficacy of tremelimumab and RT in 20 patients with human epidermal growth factor receptor normal (HER2−) BCBM. The safety of concurrent RT, tremelimumab, and trastuzumab was evaluated in a cohort of 6 HER2+ patients. The primary endpoint was 12-week non-central nervous system (CNS) disease control rate (DCR). Secondary endpoints included safety, survival, and CNS response. Exploratory correlatives included characterization of peripheral blood immune responses among exceptional responders. Tremelimumab plus RT ± trastuzumab was tolerated with no treatment-related grade 4 adverse events reported. The 12-week non-CNS DCR was 10% (2/20) in the HER2− cohort and 33% (2/6) in the HER2+ cohort. One patient with HER2+ disease experienced a durable partial response with evidence of peripheral T-cell activation. Thus, tremelimumab and RT ± trastuzumab was tolerated. Although modest clinical activity was observed in the HER2- efficacy cohort, encouraging responses were observed in the HER2+ safety cohort. Consequently, a trial to determine efficacy in HER2+ BCBM is planned.

Clinical Trial Registration Number: NCT02563925.

## Introduction

Brain metastases occur in ~30% of metastatic breast cancer patients and are increasing in incidence for patients with human epidermal growth factor 2 positive (HER2+) breast cancer^1–4^. Conventional systemic therapies have demonstrated limited effectiveness in preventing and treating breast cancer brain metastases (BCBM) at least in part because of the blood-brain barrier^5^. Thus, the cornerstone for BCBM management has been locoregional strategies such as whole brain or stereotactic radiosurgery (WBRT or SRS, respectively). However, the prognosis in this population remains poor.

Immune checkpoint inhibitor antibodies targeting programmed cell death protein 1 (PD-1) or its ligand (PD-L1) show promise in the treatment of metastatic breast cancer. Recently, pembrolizumab (an antibody targeting PD-1) was shown to improve progression free survival (PFS) and overall survival (OS) when added to chemotherapy as first-line therapy for PD-L1-expressing metastatic triple negative breast cancer^6^. However, the efficacy of anti-PD-1/L1 agents has been more modest in PD-L1-negative tumors, hormone-sensitive tumors, HER2+ trastuzumab-resistant tumors, and pre-treated advanced disease. Furthermore, data confirming benefit of anti-PD-1/L1 plus chemotherapy are lacking in patients with BCBM, as these patients are often underrepresented or excluded from clinical trials due to the poor prognosis associated with this complication and the limited brain penetrance of conventional systemic therapies.

An alternative target for immune checkpoint inhibition is cytotoxic T-lymphocyte antigen 4 (CTLA-4). Tremelimumab (AstraZeneca) and ipilimumab (Yervoy; Bristol-Myers Squibb) are both human monoclonal antibody antagonists of CTLA-4. Anti-CTLA-4 antibodies are clinically active across multiple tumor types, improve OS in metastatic melanoma, and may also enhance T-cell-mediated anti-tumor responses in breast cancer^7^. CTLA-4 is acutely upregulated following T-cell antigen exposure and activation, whereas PD-1 is upregulated following T-cell activation and sustained during chronic T-cell stimulation. In light of these mechanistic differences, anti-CTLA-4 and anti-PD-1/L1 may have differential activity across disease subtypes and clinical contexts. Recent studies have demonstrated a substantially greater effect of combination immunotherapy in multiple cancer types, highlighting the potential for combination strategies that include anti-CTLA-4 with other immune checkpoint blockers and/or standard approaches such as palliative radiotherapy(RT)^8^.

Growing evidence supports combining immune checkpoint blockade (ICB) therapy with RT in cancer. RT is associated with tumoral DNA damage, which may lead to release of antigens and danger signals that facilitate antigen presentation and tumor-specific T-cell activation, an effect that may be augmented in combination with ICB therapy^9–11^. Particularly, it has been shown that anti-CTLA-4 may inhibit T-regulatory cells and promote relative effector cell expansion, whereas the addition of RT enhances the diversity of clonal T-cell expansion, resulting in enhanced tumor regression in animal models^11^. RT may also augment immune response via upregulation of major histocompatibility complex 1 and FAS adhesion molecules^12^. Data in melanoma suggest that ICB could be safely and effectively combined with brain RT. SRS was tolerated in patients with melanoma brain metastases treated with either anti-PD-1 or anti-CTLA-4 ICB therapy^13–15^, with greater reductions in lesion volume for patients treated with ICB therapy and SRS. One study reported that SRS before or during treatment with the anti-CTLA-4 antibody ipilimumab resulted in less regional recurrence and better OS than SRS after ipilimumab^16^. Furthermore, cases of responses distant to the radiation field (the so-called abscopal effect) have been reported. However, no published studies have evaluated the efficacy of ICB (with or without RT) for the treatment of BCBM specifically.

The primary goal of this single-institution study was to determine whether anti-CTLA-4 combined with brain RT could induce systemic control of distant, non-CNS, unirradiated lesions. Thus, in an efficacy cohort, we examined the impact of concurrent tremelimumab-mediated CTLA-4 blockade with standard-of-care brain RT (either SRS or WBRT) on distant, non-CNS disease sites in patients with HER2- BCBM. Safety was evaluated in the efficacy cohort and in a separate HER2+ BCBM cohort receiving concurrent trastuzumab (Herceptin; Roche, Basel, Switzerland).

## Results

### Patients

Twenty-six patients were included in the study, 20 (77%) in the HER2- cohort and six (23%) in the HER2+ cohort. One subject from the HER2− cohort withdrew consent after receiving one dose of tremelimumab. This patient was included in the intention-to-treat analysis but had missing data for adverse events (AE)/toxicity, and laboratory studies. Patient characteristics for all participants are presented in Table 1 by cohort. Twelve out of 20 (60%) patients in the HER2− cohort and two out of six (33%) patients in the HER2+ cohort had estrogen receptor (ER)-positive disease. The median time from initial early stage diagnosis to metastatic diagnosis was 3.3 years (range: 0.0–16.3 years). The majority of patients (20/26; 76%) had metastases in 2–3 non-CNS sites and had received a median of two (range: 0–6) prior chemotherapy regimens in the metastatic setting. Patients had a median of 2.0 years (range: 0.1–7.4 years) from metastatic diagnosis to treatment start. The majority of patients (88% of all patients; 85% in HER2− cohort, 100% in HER2+ cohort) received WBRT (30 Gy/10 fractions). Among the three patients receiving SRS, doses included 21 Gy single-dose (*n* = 1 patient), hypofractionated (30 Gy/5 doses, *n* = 1 patient), or combined (21 Gy/1 fraction with 30 Gy/5 fractions, *n* = 1 patient).

**Table 1:** Patient Characteristics.

Variable	Overall *N* = 26	HER2− *N* = 20	HER2+ *N* = 6
Age, years, median (range)	50 (31–74)	51 (32–74)	41 (31–72)
Race, *n* (%)			
White	15 (58%)	14 (70%)	1 (17%)
African-American	5 (19%)	5 (25%)	0 (0%)
Asian	5 (19%)	1 (5%)	4 (67%)
Other	1 (4%)	0 (0%)	1 (17%)
ECOG status, *n* (%)			
0	11 (42%)	10 (50%)	1 (17%)
1	12 (46%)	8 (40%)	4 (67%)
2	3 (12%)	2 (10%)	1 (17%)
ER status, *n* (%)			
ER-positive	13 (50%)	12 (60%)	2 (33%)
ER-negative	13 (50%)	8 (40%)	4 (67%)
Time from initial diagnosis to metastatic diagnosis, years, median (range)	3.3 (0.0–16.3)	3.5 (0.0–12.4)	1.8 (0.1–16.3)
Time from metastatic diagnosis to study enrollment, years, median (range)	2.0 (0.1–7.4)	2.1 (0.1–7.4)	1.4 (0.5–3.4)
Metastatic non-CNS sites at study entry, *n* (%)			
Bone	21 (81%)	16 (80%)	5 (83%)
Lymph node	19 (73%)	14 (70%)	5 (83%)
Lung	20 (77%)	15 (75%)	5 (83%)
Liver	14 (54%)	12 (60%)	2 (33%)
0–1 sites	0 (0%)	0 (0%)	0 (0%)
2–3 sites	20 (76%)	15 (75%)	5 (83%)
4+ sites	6 (23%)	5 (25%)	1 (17%)
Number of prior chemotherapy regimens in metastatic setting, median (range)^a^	2 (0–6)	3 (0–6)	1 (1–5)
0 therapies, *n* (%)	3 (12%)	3 (16%)	0 (0%)
1 therapy, *n* (%)	8 (32%)	4 (21%)	4 (67%)
2 therapies, *n* (%)	2 (8%)	1 (5%)	1 (16.5%)
3 therapies, *n* (%)	7 (28%)	7 (37%)	0 (0%)
4+ therapies, *n* (%)	5 (20%)	4 (21%)	1 (16.5%)
ALC at baseline (K/µl)^a^			
<1.0	12 (48%)	8 (42%)	4 (67%)
≥1.0	13 (52%)	11 (58%)	2 (33%)
Non-CNS disease at study start, *n* (%)			
PD	25 (96%)	19 (95%)	6 (100%)
SD	1 (4%)	1 (5%)	0 (0%)
Brain RT received, *n* (%)			
WBRT	23 (88%)	17 (85%)	6 (100%)
SRS	3 (12%)	3 (15%)	0 (0%)
No. of RT doses received, median (range)	4 (1–11)	4 (1–6)	5 (2–11)

*ECOG* Eastern Cooperative Oncology Group, *HER* human epidermal growth factor receptor, *ER* estrogen receptor, *CNS* central nervous system, *ALC* absolute lymphocyte counts, *RT* radiation therapy, *WBRT* whole brain radiation therapy, *SRS* stereotactic radiosurgery.

^a^One patient not included because of consent withdrawal.

### Toxicity

Table 2 summarizes all treatment-related toxicities and details of AEs that occurred in two or more patients. Tremelimumab and RT in the HER2- cohort was tolerated, reaching pre-specified cutoffs for tolerability (see statistical methods), although treatment-related AEs were frequent (79% of patients). Serious AEs occurred in 37% of these patients, and treatment-related grade 3 AEs occurred in 32% of these patients. Concomitant tremelimumab, radiotherapy, and trastuzumab in the HER2+ cohort was also tolerated according to the pre-specified cutoffs. Treatment-related AEs occurred in 100% of HER2+ patients, with no serious AEs and one grade 3 AE (17%).

**Table 2:** Treatment-related toxicities and adverse events.

A. Treatment-related toxicities		
Events, Total (# patients)	HER2− (*n* = 19)^a^	HER2+ (*n* = 6)
AEs	104 (15)	42 (6)
SAEs	17 (7)	0 (0)
Grade 3 AEs	15 (6)	1 (1)
Grade 4 AEs	0 (0)	0 (0)
B. Treatment-related AEs in 2 or more patients		
AE, Any Grade, *n* (%)	HER2− (*n* = 19)^a^	HER2+ (*n* = 6)
Colitis	6 (32)	0
Diarrhea	6 (32)	0
Fatigue	6 (32)	3 (50)
Nausea	6 (32)	3 (50)
Anorexia	6 (32)	1 (17)
Vomiting	3 (16)	3 (50)
Weight loss	3 (16)	2 (33)
Rash, maculo-papular	3 (16)	1 (17)
Mucositis oral	1 (6)	3 (50)
Generalized muscle weakness	3 (16)	0
Abdominal pain	2 (11)	1 (17)
Dry mouth	2 (11)	1 (17)
Dysgeusia	2 (11)	1 (17)
Hypothyroidism	2 (11)	0
Headache	2 (11)	0
Alkaline phosphatase increased	2 (11)	0
Skin Hyperpigmentation	0	2 (33)
Constipation	1 (6)	1 (17)
Hyperthyroidism	1 (6)	1 (17)
Alopecia	1 (6)	1 (17)
Dry skin	1 (6)	1 (17)
Decreased T3, Free	1 (6)	1 (17)
Pruritus	1 (6)	1 (17)
AE, Grade 3, *n* (%)		
Colitis	3 (16)	0
Diarrhea	2 (11)	0
Rash maculo-papular	1 (6)	1 (17)

*HER2* human epidermal growth factor receptor 2, *AE* adverse event, *SAE* serious adverse event.

^a^One patient not included due to early consent withdrawal.

The most common treatment-related AEs of any grade that occurred in ≥10% of patients were: fatigue (46%), nausea (46%), diarrhea (24%), and colitis (24%). Treatment-related grade 3 AEs were colitis (12%) and diarrhea (8%), and all occurred in the HER2− cohort. The first of these patients was admitted to hospital for grade 3 diarrhea that persisted despite oral dexamethasone. Computed tomography (CT) imaging showed no obstruction or colitis. She was managed with IV methylprednisolone followed by infliximab, with improvement. One month later, she was readmitted with grade 3 colitis/diarrhea. CT imaging was consistent with colitis; she was managed with intravenous (IV) methylprednisolone and IVIG (intravenous immunoglobulin), and later was found to have sigmoid perforation at a site of tumor involvement and was discharged to hospice. The second of these patients was admitted with grade 3 colitis/diarrhea and colitis was confirmed by CT imaging. She was managed with IV solumedrol (2 mg/kg) with improvement in diarrhea, but was subsequently discharged to hospice for suspected progression of her liver metastases and hyperbilirubinemia. The third of these patients was admitted with grade 3 colitis, confirmed by CT imaging. She was treated with solumedrol with subsequent improvement and discharge home.

### Efficacy

Among the intention-to-treat population, 11 of the 26 patients (42% total) were not evaluable for efficacy at week 12: nine HER2− patients (45% of HER2− cohort), four due to death and five due to rapid non-CNS progression prior to week 12; and two HER2+ patients (33% of HER2+ cohort), one due to death and one due to rapid non-CNS progression prior to week 12. In the intention-to-treat HER2− cohort, the non-CNS DCR at week 12 was 10% (2/20 patients; Table 3). In the intention-to-treat HER2+ cohort, the non-CNS DCR at week 12 was 33% (2/6 patients) by RECIST 1.1 and 17% (1/6 patients) by irRC (Table 3).

**Table 3:** Responses at week 12.

A. Non-CNS responses at week 12				
Response type	HER2− (*n* = 20) RECIST 1.1	HER2− (*n* = 20) irRC	HER2+ (*n* = 6) RECIST 1.1	HER2+ (*n* = 6) irRC
DCR	2 (10%)	2 (10%)	2 (33%)	1 (17%)
CR	0 (0%)	0 (0%)	0 (0%)	0 (0%)
PR	0 (0%)	0 (0%)	1 (17%)	1 (17%)
SD	2 (10%)	2 (10%)	1 (17%)	0 (0%)
PD	9 (45%)	9 (45%)	2 (33%)	3 (50%)
Not evaluable^a^	9 (45%)	9 (45%)	2 (33%)	2 (33%)

B. CNS responses at week 12		
Response type	HER2− (*n* = 20) RANO-BM	HER2+ (*n* = 6) RANO-BM
DCR	9 (45%)	4 (67%)
CR	1 (5%)	1 (17%)
PR	2 (10%)	1 (17%)
SD	6 (30%)	2 (33%)
PD	2 (10%)	0 (0%)
Not evaluable^a^	9 (45%)	2 (33%)

*CNS* central nervous system, *HER* human epidermal growth factor receptor, *RECIST* response evaluation criteria in solid tumors, *irRC* immune-related response criteria, *DCR* disease control rate, *CR* complete response, *PR* partial response, *SD* stable disease, *PD* progressive disease, *RANO-BM* response assessment in neuro-oncology brain metastases.

^a^Due to discontinuation or death prior to evaluation at 12 weeks.

CNS response rates among the intention-to-treat population at week 12 were 15% in the HER2-negative population (*n* = 3/20; 1 CR, 2 PR), and 33% in the HER2-positive population (*n* = 2/6, 1 CR 1 PR, Table 3). Among patients who completed brain imaging at week 12 and who were evaluable by RANO criteria, the 12-week CNS response rate was 27% in the HER2− cohort and 50% in the HER2+ cohort. The 12-week CNS clinical benefit rate was 82% (*n* = 9/11) in the HER2− cohort, and 100% in the HER2+ cohort (*n* = 4/4). Median CNS-PFS could not be estimated because CNS imaging was not mandated at regular intervals following systemic progression. However, durable CNS clinical benefit was observed beyond the first CNS imaging assessment in some patients, including *n* = 4/12 patients from the HER2-negative cohort (CNS-PFS: 119d, 171d, 178 + d, 178 + d), and *n* = 4/4 patients from the HER2-positive cohort (CNS-PFS: 101 + d, 186 + d, 190 + d, 349d).

The best overall response among the 20 patients treated in the HER2- cohort was SD in two patients (10%; 95% CI: 1.2–31.7%) by both RECIST and irRC. One patient with HER2− lung and lymph node metastases had non-CNS SD at week 12 but died prior to planned confirmatory imaging at week 16. A second patient with HER2- bone, lymph node, lung, and liver metastases had SD at week 12, and PD at week 24. In addition to the SD patients described above, one patient with HER2− bone and liver metastases at study entry had PD at week 12 (24.6% increase by RECIST), SD at week 16 by RECIST (18% increase from baseline, 5.3% decrease from week 12), then PD at week 24 (34.4% increase from baseline) and died 3 months later. Following an intention-to-treat analysis and the pre-specified Simon two-stage design threshold, the DCR at week 12 in the HER2− cohort was deemed insufficient to merit further investigation.

The 12-week DCR in the HER2+ cohort was 33% (2/6). One patient with HER2+ bone and lymph node metastases had SD at week 12 (11.5% increase by RECIST, 33.8% by irRC), and PD at week 24 (44.8% by RECIST, 182.7% by irRC). One patient in the HER2+ cohort had a PR at week 12 by both RECIST and irRC that was durable at 6 months (details below).

### Survival

**Fig. 1:**
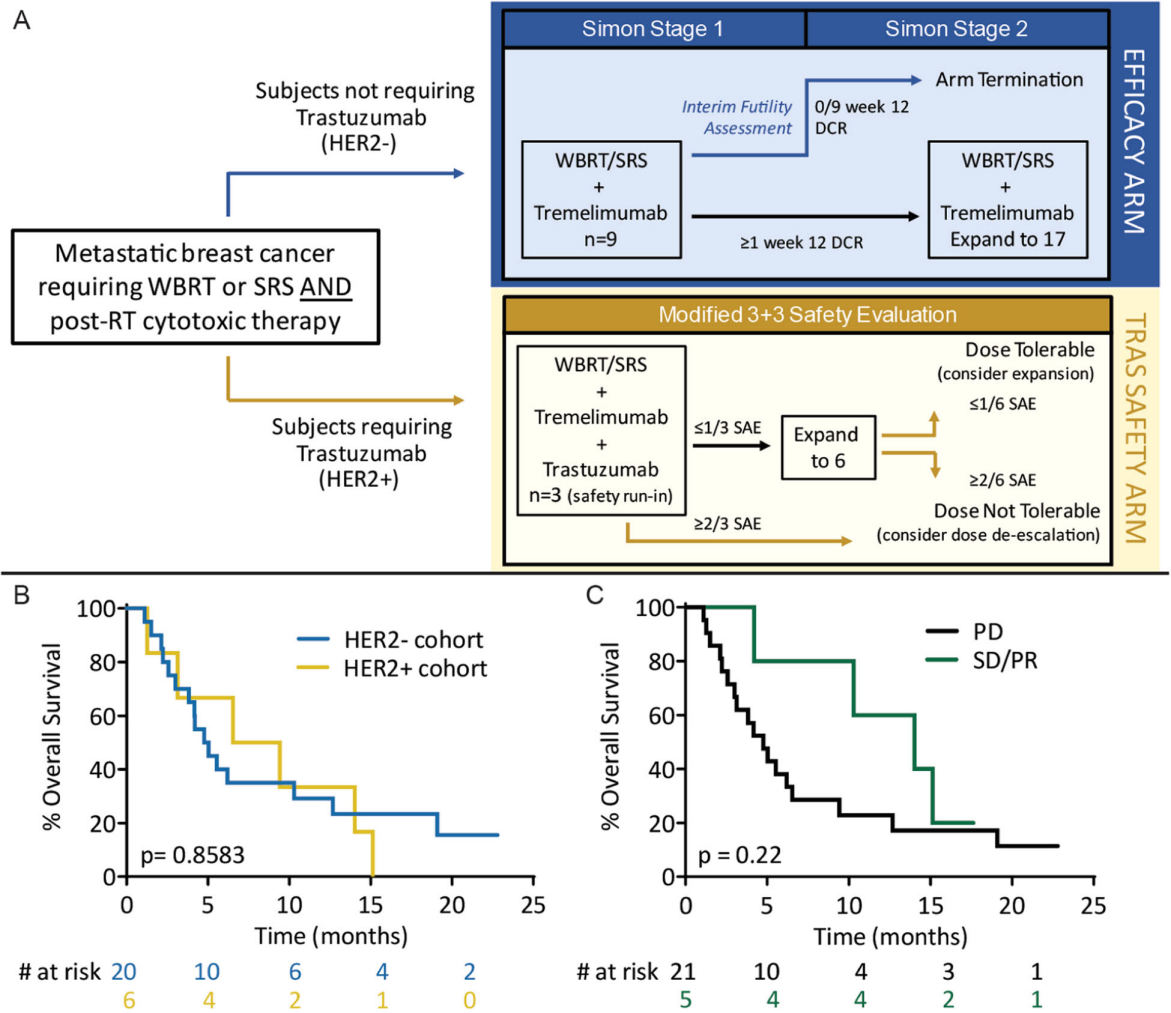
Trial schema and overall survival. **A** Trial design showing Simon two-stage design for the HER2− cohort and the safety evaluation for the HER2+ cohort. Three or more DCR responses in 17 patients at the end of the study for the treatment to be considered meritorious for further investigation. However, three additional subjects enrolled in the HER2− cohort to expand sample size for estimation of DCR at week 12. These subjects were not included in the pre-specified primary efficacy determination. **B** Median OS for HER2− cohort = 4.9 months, median OS for HER2+ cohort = 7.98 months. **C** Median OS for patients with PD = 4.47 months, median OS for patients with SD or PR = 14.58 months.

**Fig. 2:**
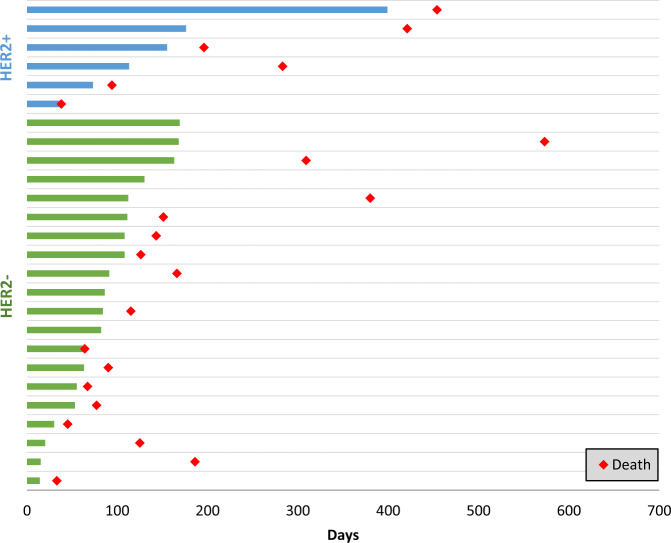
Swimmer’s plot. The Swimmer’s plot illustrates time on treatment in blue and green for the HER2+ and HER2- cohorts, respectively. Time of death is indicated by the red diamonds.

Median OS was 4.9 months for the HER2− cohort (range: 1.1–22.8+ months) and 8.0 months for the HER2+ cohort (range: 1.3–15.1) (Fig. 1B). Median PFS was 3.0 months (range: 1.1–6.2 months) for the HER2− cohort, and 3.1 months for the HER2+ cohort (range: 1.3–8.7 months). Treatment durations and times of death are illustrated in the Swimmer’s plot (Fig. 2).

### HER2+ responder: a case study

**Fig. 3:**
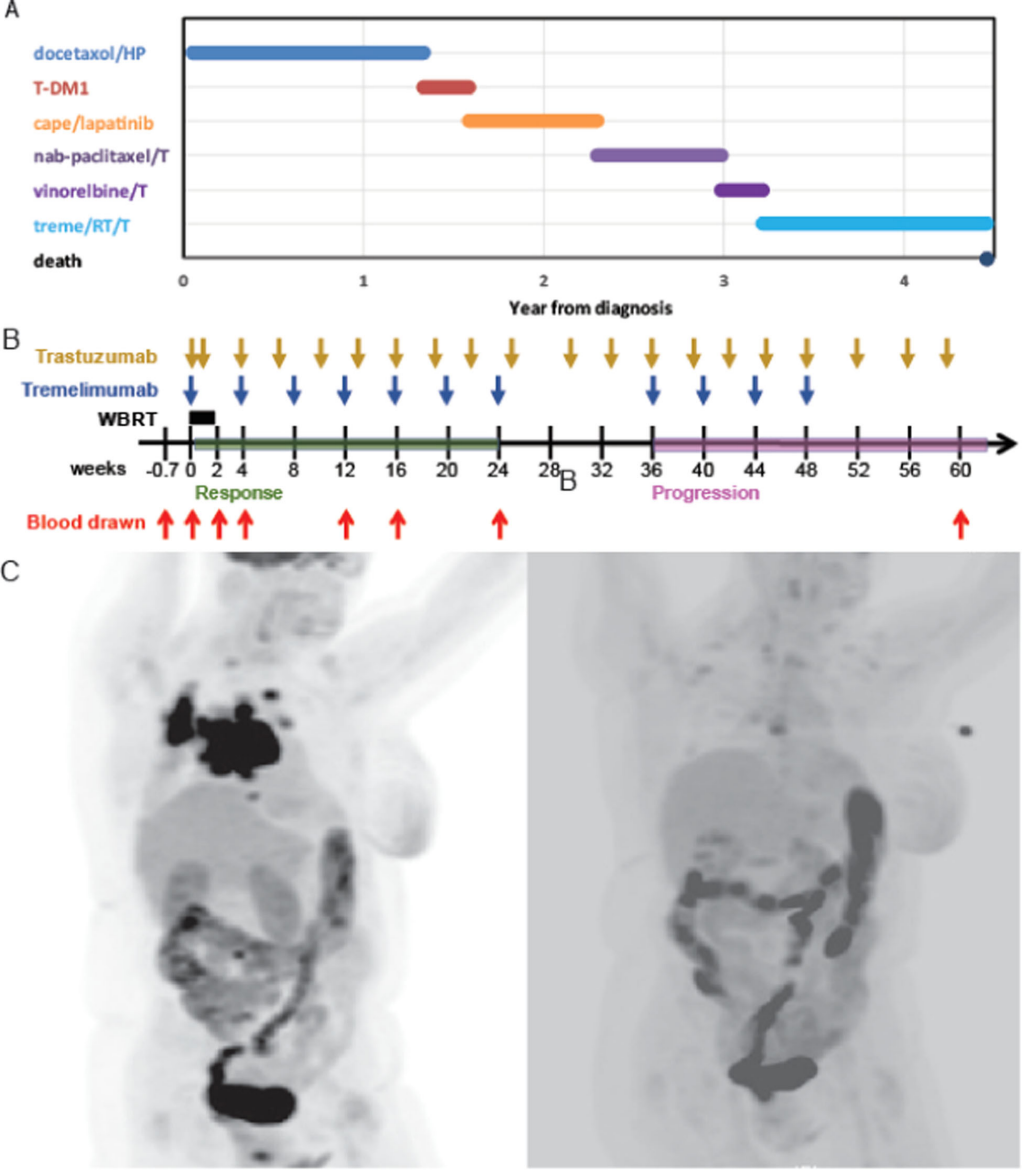
HER2+ responder treatment schedule and radiological response. **A** Prior therapies. **B** Treatment timeline and blood draws (red) for the patient who responded to tremelimumab (blue) and WBRT (black rectangle) with trastuzumab (gold), showing when she responded (green) to when she relapsed (pink). **C** Baseline (left) and week 12 (right) PET/CT demonstrating a non-CNS PR in a woman treated with concurrent tremelimumab and WBRT with HER2-directed therapy that was durable at 6 months. H trastuzumab, P pertuzumab, cape capecitabine, T-DM1 trastuzumab emtansine, T paclitaxel, treme tremelimumab, RTradiotherapy.

We observed a PR in one patient in the HER2+ cohort (Fig. 3). This patient was a 72-year-old woman who was initially diagnosed in 2012 with de novo hormone-receptor negative, HER2-positive (IHC 3+) metastatic breast cancer to mediastinal lymph nodes. She achieved a PR with first-line docetaxel/trastuzumab/pertuzumab until PD at 16 months; she received second-line trastuzumab emtansine (TDM-1) until PD at 3 months; she received third-line capecitabine/lapatinib for 8 months; then fourth-line nab-paclitaxel plus trastuzumab until PD at 6 months; and fifth-line vinorelbine plus trastuzumab until PD at 3 months (Fig. 3A). During fourth- and fifth-line therapy, the patient developed brain metastases treated with SRS to 6 areas on 2 separate occasions 5 and 7 months prior to study enrollment (i.e., a total of 12 brain metastases treated). Upon diffuse CNS progression with at least 7 new brain metastases, she enrolled in the study and received WBRT, tremelimumab, and trastuzumab (Fig. 3B). She experienced systemic PR at week 12 (56% and 86% reduction in non-CNS metastases by RECIST 1.1 and irRC, respectively) that persisted for 24 weeks (60% and 92% reduction in non-CNS metastases by RECIST 1.1 and irRC, respectively; Fig. 3C). She received trastuzumab alone between weeks 24 and 36 per protocol. At week 36 she had ongoing disease control of her mediastinal disease (58% reduction from baseline by RECIST 1.1) but developed new skin lesions that were biopsy-proven HER2+ breast cancer metastases. Despite the history of multiple recurrences of brain metastases the year prior to study enrollment, this patient experienced a durable CNS CR throughout the study period and until month 12.

**Fig. 4:**
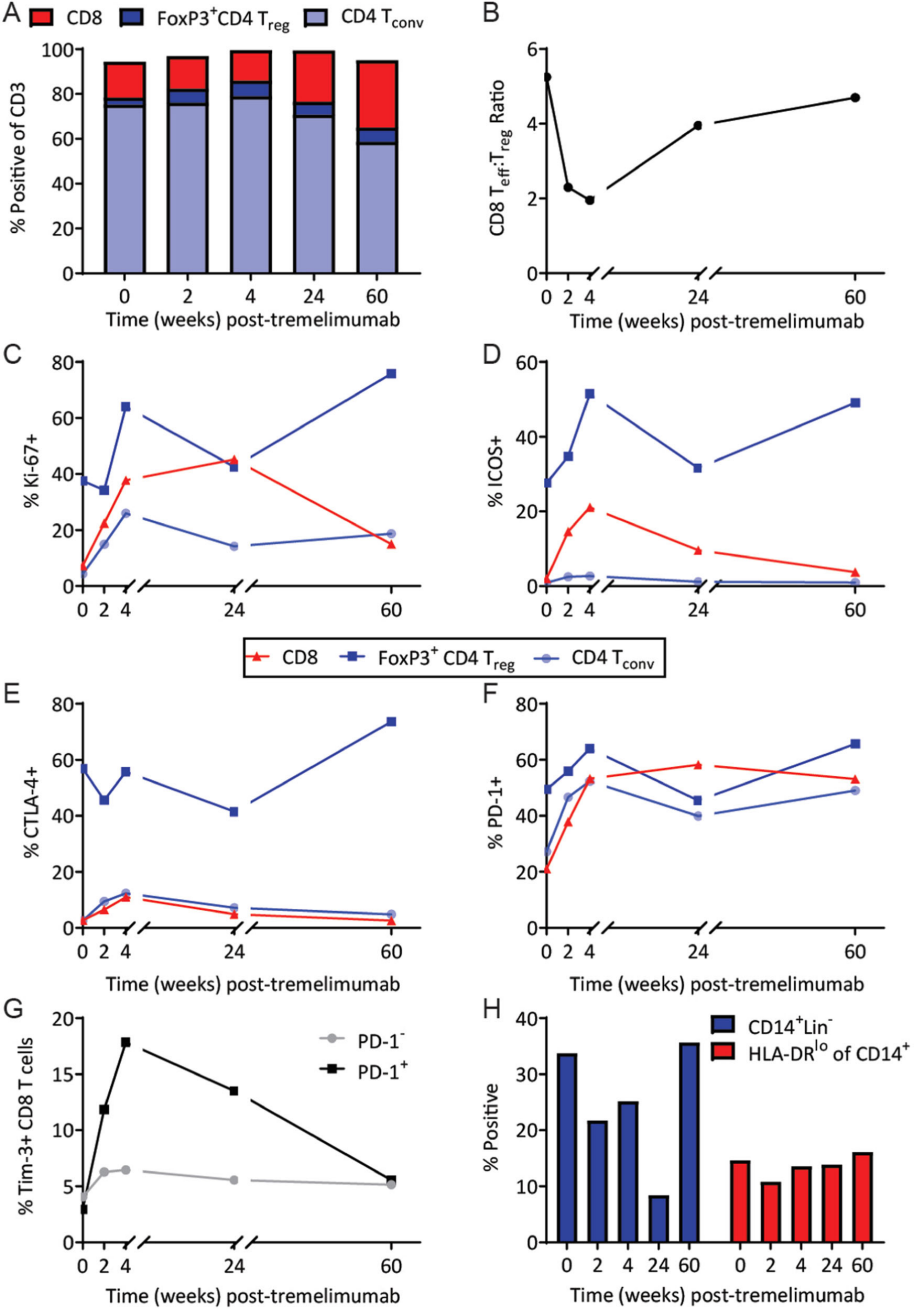
Immunologic correlates in the blood of a HER2+ responder. Peripheral blood mononuclear cells were stained for various markers and analyzed using flow cytometry at weeks 0, 2, 4, 24, and 60 post-treatment initiation (*n* = 2, technical replicates). **A** Expression of CD8 (red), FoxP3^−^CD4 conventional T cells (T_conv_; light blue), and FoxP3^+^CD4 regulatory T cells (T_reg_; dark blue) out of total CD3^+^ live cells. **B** CD8:T_reg_ ratio. (C-F) CD8 (red triangles), CD4 T_conv_ (light blue circles), and T_reg_ (dark blue squares) were analyzed for expression of (**C**) Ki-67, (**D**) ICOS, (**E**) CTLA-4, and (**F**) PD-1. **G** Tim-3 expression on PD-1^+^ compared to PD-1^−^ CD8 T cells. **H** Percentage of CD14^+^ and HLA-DR_lo_ monocytes over time.

PBMCs and serum cytokines were analyzed by flow cytometry at several time points during the course of treatment. Over the 60-week follow-up period, there was a slight decrease in the frequency of FoxP3^−^ CD4 T conventional (T_conv_) cells, increase in CD8 T cells, and minimal change in FoxP3^+^ CD4 T-regulatory (T_reg_) cells (Fig. 4A). We observed a decrease in the CD8 T_eff_:T_reg_ ratio in the first few weeks after the start of treatment, followed by an increase at weeks 24 and 60 (Fig. 4B). Several groups have proposed T-cell activation markers, including ICOS and Ki-67, as pharmacodynamic biomarkers in the context of anti-CTLA-4^17–19^. In this patient, Ki-67 and ICOS expression in CD4 T_conv_ and T_reg_ cells increased following treatment initiation and subsequently declined by week 24 (Fig. 4C, D).

We also measured expression of checkpoint molecules CTLA-4 and PD-1 (Fig. 4E, F). CTLA-4 expression on CD4 T_conv_ and CD8 T cells transiently increased through week 4 and then returned to baseline; CTLA-4 expression in T_reg_, which is expected to be higher than in conventional T cells, remained elevated with no clear trend over time. For CD4 T_conv_ and CD8 T cells, PD-1 expression peaked at week 4 and remained elevated through week 60; for T_reg_, PD-1 expression peaked at week 4, returned to baseline at week 24, and then peaked again at week 60. Interestingly, we observed a transient increase in the frequency of circulating Tim-3^+^ PD-1^+^ CD8 T cells, whereas Tim-3 expression was absent in the PD-1^−^ CD8 T-cell population (Fig. 4G). We also evaluated peripheral CD14^+^ monocytes and HLA-DR^lo^CD14^+^ MDSCs (Fig. 4H). The frequency of CD14^+^ monocytes declined through week 24 and then returned to baseline by week 60; MDSCs remained stable throughout the course of treatment. TCR sequencing of PBMCs from this patient revealed that treatment was associated with the emergence of a substantial number of low-abundance clonotypes (Supplementary Fig. 1). This effect persisted while on therapy until the patient experienced progression at week 60. Therapy was not associated with the emergence of dominant clones or expansion of previously existing dominant clones.

Relative to preceding timepoints, at the time of tumor progression (Week 60 timepoint), activation markers and immune checkpoints were highly expressed in T_reg_ cells, but relatively decreased in effector cells (CD4 and CD8). At this timepoint, we also observed clonal T-cell hyperexpansion and an increase in the effector/regulatory T-cell ratio, highlighting the possibility that immune responses against the progressive skin metastases were present but non-productive, and perhaps impaired by T_reg_ cells and MDSCs.

## Discussion

Tremelimumab plus CNS RT with or without trastuzumab was tolerated in metastatic breast cancer. Several subjects experienced severe immune-related toxicities consistent with known class effects of anti-CTLA-4 therapy, which included diarrhea, colitis, and rash. In all but one case, these toxicities were effectively managed with established management algorithms that employ supportive care, corticosteroids and other immune-modulatory agents. One subject experienced colon perforation following a course of immune-related colitis, however the patient also suffered from sepsis and intra-abdominal metastases.

Tremelimumab with CNS RT demonstrated inadequate activity to justify further study in the HER2- efficacy cohort and several factors may have contributed to this outcome. First, most patients in this cohort had hormone-receptor positive breast cancer, which are known to be less responsive to immune checkpoint therapy relative to triple negative breast cancer for a variety of proposed reasons, including low tumor mutational burden and suppressive transforming growth factor beta signaling within the microenvironment. For example, no objective responses were observed in a previous trial of tremelimumab plus exemestane in hormone-receptor positive disease and responses to anti-PD-1/L1 in advanced hormone-receptor positive breast cancer have been similarly modest^20,21^. Second, several subjects died of disease progression after just one dose of therapy, highlighting the unique challenges of clinical investigation in this poor prognosis population. Third, subjects in this trial were heavily pre-treated with cytotoxic therapy, which may diminish likelihood of response to immune checkpoint therapy. For example, reported responses to anti-PD-1/L1 monotherapy are 21–26% in the first-line setting for triple negative metastatic breast, but only 5–11% in later lines^22–25^. The diminished success of immune checkpoint later in the course of the disease may be explained by host factors such as chemotherapy-related lymphopenia and impaired functional status, and/or tumor factors such as tumor burden, direct immunosuppressive effects, and/or immune evasion.

Despite the above barriers to response, one heavily pre-treated subject with HER2+ disease experienced a durable (36 week) systemic PR following treatment tremelimumab, trastuzumab, and WBRT. Because the disease was trastuzumab-refractory, we attribute the systemic response to an interaction effect involving tremelimumab with trastuzumab and/or radiotherapy. Trastuzumab is known to stimulate T-cell anti-tumor immunity^26^, facilitate antibody-dependent cellular cytotoxicity via natural killer cells, and augment cross-presentation of tumor antigens via dendritic cells^27^. RT has been shown in numerous animal models to synergize with anti-CTLA-4. In metastatic melanoma, combined anti-CTLA-4 (ipilimumab) and RT resulted in favorable response rates and survival relative to prior reports of ipilimumab alone^28–31^. Moreover, RT combined with ICB may increase the frequency of systemic, or ‘abscopal’, responses^31–33^. Finally, trastuzumab has also been shown to radiosensitize cancer cells in vitro, suggesting synergy of radiotherapy and trastuzumab^34,35^. We propose that concurrent administration of RT with trastuzumab may radiosensitize tumors and induce immunogenic cell death, while facilitating antigen presentation via and allowing for T-cell activation that is enhanced by concomitant tremelimumab.

Peripheral blood immune monitoring of this responding patient provided insight on the underlying immune response. In previous trials, peripheral markers of T-cell activation (such as ICOS and/or Ki-67) were found to be predictive of anti-CTLA-4 response and improved survival^17–19^. In the HER2+ responder, we observed increases in ICOS and Ki-67 in CD4 and CD8 effector cells. These findings support our assertion that the observed durable response was related to T-cell activation. We also identified therapy-associated upregulation of several immune checkpoints including CTLA-4, PD-1, and Tim-3, which may reflect acute T-cell activation, but also underscores the potential for subsequent T-cell exhaustion/dysfunction and tumor immune escape. For example, previous studies have shown that Tim-3 and PD-1 expression on CD8 T cells can indicate early T-cell activation, but also exhaustion/dysfunction in the setting of RT^36–38^. These findings highlight an opportunity to investigate anti-Tim-3 in combination with RT and other immune checkpoint antibodies in breast cancer and in other malignancies^39^.

The potential role of tremelimumab/RT should be considered in the context of recent therapeutic advances for HER2+ BCBMs. Recently, small molecule inhibitors of the HER2 protein (for example, tucatinib and neratinib) have been shown to penetrate the blood-brain barrier and exhibit clinical activity in the context of active HER2+ BCBMs^40,41^. Similarly, antibody-drug conjugates such as ado-trastuzumab emtansine and fam-trastuzumab deruxtecan have been approved by the United States Food and Drug Association for treatment of HER2+ metastatic disease, and CNS activity has been reported^21,42^. In the PANACEA study, pembrolizumab plus trastuzumab was found to be clinically active, however responses were restricted to PD-L1-positive tumors, and patients with known CNS disease were excluded^43^. Because many patients will experience CNS recurrence or progression, tremelimumab/trastuzumab could be an effective strategy for patients who are indicated to receive brain RT. Furthermore, anti-CTLA-4-based combinations may be uniquely suited to benefit patients with advanced PD-L1-negative disease, who are unlikely to benefit from anti-PD-1/L1 based therapy. Therefore, tremelimumab/trastuzumab could fill an unmet clinical need, and merits more definitive clinical evaluation to confirm clinical efficacy and safety.

Because of the single-institution, non-randomized nature of this trial, future investigation is planned to confirm efficacy in the treatment of HER2+ BCBM and to address other inherent limitations of the data. Preclinical studies suggest that radiation modality, dose, fractionation, and sequencing (with respect to immunotherapy) may be relevant biologic response modifiers^44–46^, and thus various sequencing and dosing approaches must be compared. For example, higher RT doses may be associated with more profound release of damage associated molecular patterns (DAMPs, including ATP and HMGB1), and greater extent of synergy with ICBs^47–49^. In this trial, 88% of subjects received WBRT, therefore in future trials, WBRT in conjunction with SRS (increased RT dose) should be considered as a method to maximize immunogenic effects of RT when combined with tremelimumab or other ICBs. Also, future evaluation of lower-dose anti-CTLA-4 may be considered to mitigate toxicity, potentially in combination with anti-PD-1/L1, which was shown in a melanoma model to enhance response of anti-CTLA-4 + RT^11^.

Thus, tremelimumab and brain RT was tolerated but associated with reversible immune-mediated grade 3 toxicities attributed to anti-CTLA-4 class effect. The regimen was not sufficiently active to merit further study in HER2− disease. However, stable disease and a partial response lasting >6 months were observed in patients with trastuzumab-refractory advanced HER2+ disease receiving tremelimumab and brain RT plus trastuzumab. In light of this data, further clinical study to confirm activity of checkpoint inhibition and RT plus trastuzumab in HER2+ breast cancer is planned.

## Methods

### Study design and patient selection

Between September 2015 and May 2016, breast cancer patients with measurable non-CNS disease and brain metastasis for whom standard-of-care brain radiotherapy (either SRS or WBRT) was planned were enrolled at Memorial Sloan Kettering Cancer Center. Inclusion criteria included: age ≥18 years, Eastern Cooperative Oncology Group status of 0–2, life expectancy ≥12 weeks, pathologically-confirmed invasive breast carcinoma of any histologic subtype, radiologically-confirmed brain metastases of any histology, and Response Evaluation Criteria In Solid Tumors (RECIST 1.1) measurable non-central nervous system (CNS) metastatic disease for which a change in systemic therapy was planned. Exclusion criteria included CNS complications requiring urgent neurosurgical intervention or intrathecal therapy for leptomeningeal disease. Patients with ongoing reversible National Cancer Institute Common Terminology Criteria for Adverse Events (CTCAE v4.0) greater than grade 2 chemotherapy-related toxicities were also excluded (NCT02563925 registered 9/29/15).

Participants were assigned to cohort A (HER2−) or cohort B (HER2+), depending on indication for ongoing trastuzumab therapy (Fig. 1A). Twenty patients were enrolled in the HER2− cohort, following a Simon two-stage statistical design to assess efficacy. Specifically, an assessment was conducted after the first 9 subjects were enrolled, and accrual to this arm proceeded after the pre-specified futility threshold (≥1/9 with 12-week non-CNS disease control) was met.

Participants with HER2+ disease, defined as either 3+ expression by immunohistochemistry and/or >2.0 *HER2*/chromosome 17 centromere signals by fluorescence in situ hybridization, were enrolled to safety cohort B. HER2+ cohort subjects were treated with tremelimumab and brain radiotherapy plus trastuzumab. The primary objective for this cohort was to evaluate the safety of the combination. A safety run-in of three patients was performed before expanding the cohort to six patients after meeting a pre-specified threshold (≤1/3 experiencing a serious AE, defined in safety assessment section).

### Treatment interventions

For all patients, 10 mg/kg intravenous (IV) tremelimumab (MedImmune, Mountain View, CA) was administered within 5 days prior to or 3 days after the first fraction of radiotherapy. Subsequent doses were administered every 28 days ± 1 week for 6 months, then every 3 months, until disease progression or until treatment was not tolerated. Patients in the HER2+ cohort received maintenance trastuzumab at 2 mg/kg IV weekly or 6 mg/kg IV every three weeks, per the treating doctor’s discretion. Cardiac monitoring with transthoracic echocardiogram was conducted every 12 weeks for patients receiving concurrent trastuzumab.

Patients received standard-of-care WBRT or SRS, as determined by the treating radiation oncologist. WBRT was administered over 10 days in fractions of 3 Gy, with right and left lateral equally weighted fields, using a megavoltage linear accelerator with 6MV or higher. The dose was calculated on the central ray at mid-separation of the beams. SRS was administered in cases of oligometastatic disease. Dosing was dependent on several factors including lesion size and location, but was typically delivered in one fraction. This study was approved by the Memorial Sloan Kettering Institutional Review Board and conducted in accordance with the Declaration of Helsinki. All patients provided informed consent.

### Clinical trial objectives

The primary objective of the HER2− cohort was to evaluate the non-CNS DCR at 12 weeks following treatment with tremelimumab and brain radiotherapy (SRS or WBRT). When clinically feasible, subjects with progressive disease (PD) at week 12 continued treatment on study and were re-evaluated at week 16 to evaluate for delayed clinical response (i.e., rule out pseudoprogression). The primary objective of the HER2+ cohort was to evaluate the safety of concurrent trastuzumab plus brain radiotherapy and tremelimumab. Secondary objectives were to evaluate: non-CNS response rates by immune-related response criteria (irRC);^50^ rates of CTCAE toxicities following tremelimumab and trastuzumab; and PFS, OS, and RECIST/irRC response rates following tremelimumab and radiotherapy with trastuzumab. Response Assessment in Neuro-Oncology Brain Metastases was used to evaluate the brain metastases^51^.

### Safety assessment

Patients were followed with regular physical examinations and toxicity assessment using CTCAE v4.0. In the HER2− cohort, a continuous safety assessment was conducted using a repeated significance testing method to prevent excess exposure to undue and unexpected toxicities^52^. For ascertainment of the primary endpoint, serious AEs were defined as any of the following: death; a life-threatening AE; an AE that results in inpatient hospitalization or prolongation of existing hospitalization; a persistent or significant incapacity or substantial disruption of the ability to conduct normal life functions; a congenital abnormality or birth defect; or a medical event that requires medical/surgical intervention to prevent another serious outcome listed in this definition.

### Pharmacodynamic evaluation

To aid in hypothesis generation of immunologic mechanisms of response, immune monitoring was conducted on peripheral blood mononuclear cells (PBMCs) collected from responding patients, using a comprehensive panel of assays offered by the MSKCC immune monitoring core facility, including multiplexed flow cytometry, multiplexed serum cytokine assay, and T-cell receptor quantitative sequencing. Human PBMC samples were thawed and stained with a fixable Aqua viability dye (Invitrogen) and a cocktail of antibodies to the following surface markers: CD8-Qdot605 (Invitrogen, 3B5), CD4-Qdot 655 (Invitrogen, S3.5), PD-1-PE (BD, MIH4), LAG-3-FITC (Enzo, 17B4), ICOS-PE-Cy7 (eBioscience, ISA-3), TIM-3-APC (R&D Systems, 344823). Cells were next fixed and permeabilized with the FoxP3/Ki-67 Fixation/Permeabilization Concentrate and Diluent (eBioscience), and subsequently stained intracellularly with CD3-BV570 (Biolegend, UCHT1), Ki-67-AlexaFluor700 (BD, B56), FOXP3-eFluor450 (eBioscience, PCH101), and CTLA-4-PerCP-eFluor710 (eBioscience, 14D3). Stained cells were acquired on a BD Biosciences LSRFortessa and analyzed using FlowJo software (FlowJo, LLC). A second flow panel was used to evaluate the impact of treatment on a subset of immune cells classified phenotypically as peripheral myeloid-derived suppressor cells (MDSCs). They were characterized by staining human PBMC with: Live/Dead Aqua Fixable viability stain (ThermoFisher Scientific, Waltham, MA), an exclusion lineage cocktail (CD3/CD16/CD19/CD20/CD56)-FITC (BD Pharmingen), CD14-PerCP-Cy5.5 (BD Pharmingen), and HLA-DR-ECD (Beckman Coulter, Pasadena, CA). Samples were analyzed using an LSRFortessa flow cytometer with FACSDiva (BD Biosciences) and FlowJo software (TreeStar, Inc., Ashland, OR). The MSD V-PLEX Human Proinflammatory Panel multiplex cytokine immunoassay kit (Meso Scale Discovery, Rockville, MD) was used following manufacturer instructions to quantify serum concentrations of cytokines during treatment for the HER2+ responder, including: IFN-γ, IL-1β, IL-2, IL-4, IL-6, IL-8, IL-10, IL-12p70, IL-13, and TNF-α. PBMCs were analyzed for T-cell diversity and clonality by T-cell receptor DNA deep sequencing using the ImmunoSEQ assay^53^.

### Statistical analysis

A Simon two-stage design for the HER2− cohort tested the null hypothesis that the true non-CNS disease control rate was ≤5% versus the alternative hypothesis that the true non-CNS disease control rate was >25% or higher. This design used a Type I error rate of 5% and had 80% power to reject the null hypothesis if the true non-CNS disease control rate was 25%. This design required one or more DCR responses in nine patients at the end of stage one to proceed to stage two, and three or more DCR responses in 17 patients at the end of the study for the treatment to be considered meritorious for further investigation. The point estimate and 95% confidence intervals were reported for efficacy outcomes.

All patients who received at least one dose of tremelimumab were included in both the safety and efficacy intention-to-treat analyses. However, three additional subjects enrolled in the HER2− cohort to expand sample size for estimation of DCR at week 12. These subjects were not included in the pre-specified primary efficacy determination.

The proportion of patients with non-CNS week 12 DCR (CR + PR + SD) and overall best response by RECIST and irRC was estimated. OS was defined as the time from the first dose of tremelimumab until death from any cause. PFS was defined as the time from the first dose of tremelimumab until non-CNS progression by RECIST. OS and PFS were assessed using Kaplan–Meier analysis. Data were analyzed and graphed using Prism 8 (GraphPad, La Jolla, CA).

### Reporting summary

Further information on research design is available in the Nature Research Reporting Summary linked to this article.

## Supplementary information

Reporting Summary Checklist

Supplemental figure 1

## Data Availability

The data that support the findings of this study are available from the corresponding author upon reasonable request.
