# Supplementary material for: Brain radiotherapy, tremelimumab-mediated CTLA-4-directed blockade +/− trastuzumab in patients with breast cancer brain metastases
Source: NPJ Breast Cancer. 2022 Apr 19;8:50. doi: 10.1038/s41523-022-00404-2 (PMC9018738; doi:10.1038/s41523-022-00404-2)
Supplement: Supplementary file 2 — Supplemental figure 1 [file 41523_2022_404_MOESM2_ESM.pdf]

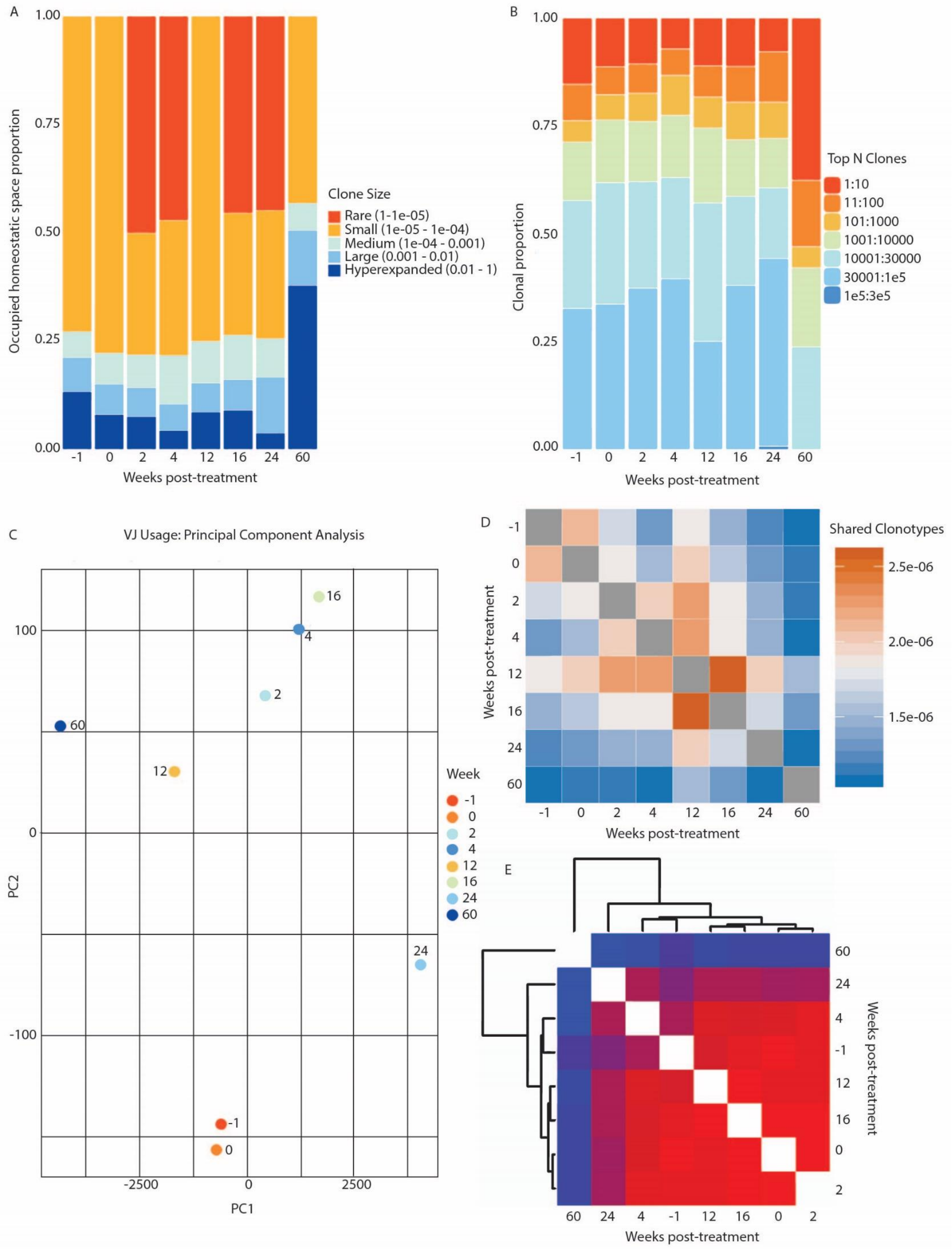

A
Occupied homeostatic space proportion
Weeks post-treatment
Clone Size
Rare (1-1e-05)
Small (1e-05 - 1e-04)
Medium (1e-04 - 0.001)
Large (0.001 - 0.01)
Hyperexpanded (0.01 - 1)
B
Clonal proportion
Weeks post-treatment
Top N Clones
1:10
11:100
101:1000
1001:10000
10001:30000
30001:1e5
1e5:3e5
C
VJ Usage: Principal Component Analysis
PC1
PC2
Week
D
Shared Clonotypes
2.5e-06
2.0e-06
1.5e-06
Weeks post-treatment
E
Weeks post-treatment

Supplemental Figure 1. Peripheral blood T-cell repertoire changes in a HER2+ breast cancer brain metastasis patient responding to tremelimumab, trastuzumab, plus radiotherapy.

Legend: A-B. Therapy was associated with an increase in low-prevalence clonotypes, whereas at progression there was evidence of clonal hyper-expansion; C. Therapy was associated with repertoire remodeling, indicated by clustering of pre-treatment versus post-treatment specimens (principal component analysis) as well as D. higher clonotype overlap. E. Unsupervised cluster analysis according to timepoint (red indicates greater overlap in repertoire).
